# Draft Genome Sequence of *Helicobacter* sp. Strain CaF467b, Isolated from a Pig Manure Storage Tank

**DOI:** 10.1128/mra.00255-22

**Published:** 2022-07-11

**Authors:** Galen Guo, Wen Chen, Michel Cloutier, John Chmara, Suzanne Gerdis, Julie Chapados, Jeremy Dettman, Izhar U. H. Khan

**Affiliations:** a Ottawa Research & Development Centre, Science & Technology Branch, Agriculture and Agri-Food Canada, Ottawa, Ontario, Canada; Loyola University Chicago

## Abstract

In this report, we present the draft genome sequence of an unclassified *Helicobacter* strain, CaF467b. This bacterial isolate was recovered from a pig manure storage tank. The draft genome sequence is 1,655,514 bp in length with 1,709 predicted genes and a G+C content of 34.07%.

## ANNOUNCEMENT

*Helicobacter* species are Gram-negative, curved bacilli commonly found in the gastrointestinal environment of humans (1, 2) and animals (3, 4). Recently, non-Helicobacter pylori species have been considered emerging human pathogens since they have been reported in clinical cases (5–7) that are often acquired through direct contact with infected animals (8). Therefore, there is an urgent need to understand the full nature of their pathogenesis.

Strain CaF467b was recovered from a pig manure storage tank located in Ayer’s Cliff, Quebec, Canada. The sample was aseptically collected in a sterile bottle containing was processed 1× phosphate-buffered saline (PBS). The suspension was 10-fold serially diluted and isolated on modified Karmali agar (Thermo Fisher Scientific, KS, USA) containing antimicrobial supplements under microaerophilic (5% O_2_, 85% N_2_, 10% CO_2_) conditions for 48 h. DNA was extracted from a purified colony using a DNeasy UltraClean microbial kit (Qiagen, MD, USA) as per the manufacturer’s instructions. Initial 16S rRNA amplicon sequencing, using the primer pair pA-F (5′-AGAGTTTGATCCTGGCTCAG-3′) and pH-R (5′-AAGGAGGTGATCCAGCCGC-3′), showed that strain CaF467b had the highest sequence identity to Helicobacter canadensis strain MIT 98-5491 (Accession number GCF_000162575.1).

Libraries were prepared and pooled using the DNA prep and NextSeq 500 mid output reagent kits (Illumina, CA, USA). Whole-genome sequencing was performed at the AAFC Ottawa Research and Development Centre on the Illumina NextSeq 500 platform (2 × 150 bp). We obtained a total of 1,826,288 paired-end raw reads; adapters and low-quality bases were trimmed using fastp v.0.20.1 (9), and the cleaned paired-end reads were assembled using Megahit v.1.2.9 (10), where sequences of >5,000 bp were excluded. The taxonomy was estimated using the *anvi-run-scg-taxonomy* function from Anvi’o v.7.1 (11) against the GTDB release 202 (12). Assemblies were refined such that 20 contigs were identified as *Helicobacter* using *anvi-refine* from Anvi’o (11). CheckM v.1.1.3 (13) was used to assess the quality of the final draft genome of strain CaF467b, which showed a completeness of 99.71% with 0.35% contamination. The whole-genome alignment and assembly were improved using RagTag (14, 15), with *H. canadensis* (GCF_000162575.1) as the reference genome as determined by average nucleotide identity (ANI) analysis (1).

The draft genome is 1,655,514 bp long, with an average coverage of 119.98×, an *N*_50_ value of 116,880 bp, and a G+C content of 34.07%. The ANI was calculated using MinHash (16) through GTDB-tk (17). ANI analysis showed that this draft genome has a 90.3% similarity to the reference genome of *H. canadensis* (GCF_000162575.1) and 98.8% similarity to the draft genome of *Helicobacter* sp. strain 11-8110 (GCF_003288905.1). Annotation using the Prokaryotic Genome Annotation Pipeline (PGAP) yielded a total of 1,653 coding genes, 18 pseudogenes, and 35 tRNA genes (18). Using ABRicate v.1.0.1 (19), we screened for potential antibiotic resistance genes and detected *tetW*, with a coverage of 85.5% and identity of 99.82% against the NCBI AMRFinderPlus database (20) and a coverage of 85.52% and identity of 98.72% against the CARD database (21).

The draft genome sequence reported here could serve as a reference genome for comparative genomics, expand the taxonomic branch of other non-H. pylori *Helicobacter* spp., and be mined for zoonotic pathogenic potential.

### Data availability.

The whole-genome shotgun project for strain CaF467b has been deposited at DDBJ/ENA/GenBank under the accession number JALAIY000000000.1 and under the BioProject accession number PRJNA809382. The version described in this paper is version JALAIY010000000. The raw sequencing files can be found under the SRA accession number SRR18128650.
